# Regulation of early diagnosis and prognostic markers of lung adenocarcinoma in immunity and hypoxia

**DOI:** 10.1038/s41598-023-33404-8

**Published:** 2023-04-20

**Authors:** Kang Sun, Zhiqiang Zhang, Dongqin Wang, Yinlong Huang, Jing Zhang, Chaoqun Lian

**Affiliations:** 1 Research Center of Clinical Laboratory Science, Bengbu Medical College, Bengbu, 233030 China; 2 Department of Biochemistry and Molecular Biology, School of Laboratory Medicine, Bengbu Medical College, Bengbu, 233030 China; 3 Department of Genetics, School of Life Sciences, Bengbu Medical College, Bengbu, 233000 China

**Keywords:** Cancer, Cell biology, Genetics, Immunology, Health care, Health occupations

## Abstract

Lung adenocarcinoma is still cancer with the highest mortality. Hypoxia and immunity play an essential role in the occurrence and development of tumors. Therefore, this study is mainly to find new early diagnosis and prognosis markers and explore the relationship among the markers and immunity and hypoxia, to improve the prognosis of patients. Firstly, based on the clinical database in TCGA, we determined the most critical clinicopathological parameters affecting the prognosis of patients through a variety of analysis methods. According to pathological parameters, logistic most minor absolute contraction selection operator (lasso), univariate and multivariate regression analysis, the risk genes related to early prognosis were screened, and the risk model was established. Then, in different risk groups, GSEA and CIBERSORT algorithms were used to analyze the distribution and enrichment of the immune cells and hypoxia, to study the effects of early prognostic indicators on hypoxia and immunity. At the same time, we analyzed the different levels of risk genes in normal cells (BSEA-2B) and tumor cells (H1299, A549, PC9, and H1975). Finally, A549 and PC9 cells were induced by CoCl2 to establish a hypoxic environment, and the correlation between risk genes and HIF1A was analyzed. The risk model based on risk genes (CYP4B1, KRT6A, and FAM83A) was accurate and stable for the prognosis of patients. It is closely related to immunity and hypoxia. In BSEA-2B cells, the mRNA and protein expression of CYP4B1 was higher, while the expression of KRT6A and FAM83A was lower. Finally, we found that FAM83A and HIF1A showed a significant positive correlation when A549 and PC9 cells were exposed to hypoxia. The discovery of early diagnostic markers related to immunity, hypoxia, and prognosis, provides a new idea for early screening and prognostic treatment of lung adenocarcinoma.

## Introduction

Lung cancer is a disease with the highest mortality among all cancers, and non-small cell lung cancer accounts for about 85% of lung cancer^1^. Lung adenocarcinoma is the most common histological category of non-small cell lung cancer; It is rising among younger men and women^2,3^. Although surgery and combination therapy have been improving in recent years, the five-year survival rate of patients is still less than 20%^4–8^. Through a variety of modern methods, the prognosis of patients can be diagnosed and analyzed. Therefore, the TMN Stage has a higher five-year survival rate than other Stages^9^. Therefore, finding new early molecular prognostic markers can quickly and accurately determine the Stage of TMN, which is a very profitable strategy for patient treatment. Identifying the PD-1/PD-L1 immune checkpoint pathway as a therapeutic target for inducing an immune response has shown excellent prospects to respond to tumor cells^10^. However, this treatment is effective only for some (20–30%) patients^11–14^. At the same time, in most solid tumors, hypoxia is a crucial factor to promote the survival and adaptation of tumor cells and help cancer cells progress^15^. Therefore, Therefore, the development of new early prognostic markers related to immunity and hypoxia may improve the prognosis of patients with lung adenocarcinoma.

In this study, early diagnostic markers were screened using the gene transcriptome of the regular group and TMN Stage and the protein database of CPTAC, respectively. Then, risk genes were filtered from the early diagnosis and prognostic markers (edgps), using various statistical methods. At the same time, we also discussed the relationship between the risk model and immunity, hypoxia, mutation, and clinical prognosis. Finally, we studied the distribution difference of risk genes between tumor cells and normal cells and the correlation between HIF1A and risk genes in a hypoxia environment. In conclusion, this series of findings will provide ideas and strategies for patients' prognosis and early diagnosis.

## Materials and methods

### Data source

We downloaded the data sets of lung adenocarcinoma-related proteins and genes, including Cancer Genome Atlas (TCGA), Gene Expression Omnibus (GEO), and clinical proteomic Tumor Analysis Consortium. The gene transcription RNA-seq data and clinical information of 59 normal and 535 tumor patients, were downloaded from the TCGA database. The GEO (GSE26939) and CPTAC datasets contained 113 columns of clinical patient information, 102 normal, and 109 tumor proteomic data, respectively.

### Screening of early diagnostic genes

We divided the RNA-seq data obtained from TCGA into two groups, including Stage I-normal and Stage I-Stage II-IV, which were analyzed for differential expression by “limma” package in R language; the screening criteria was |logFC|> 1 and 0.5, FDR < 0.5, respectively. Then, differential protein data was obtained through the normal and tumor groups of CPTAC and limma in R language; the screening criteria were |logFC |> 0.5, *p *value < 0.05. Three groups of differentially expressed genes and proteins were intersected to obtain early diagnostic genes (edgs).

### Screening early diagnosis and prognosis genes and establishing early prognosis risk model

Firstly, after screening and dimensionality reduction of the early diagnosis and prognosis gene (edgps) through univariate and lasso Cox regression analysis, risk genes can be obtained. Then, after multivariate Cox regression analysis, the risk coefficient of risk genes (rgs) can be calculated to establish the early prognostic risk model (edgpsl). Finally, the calculation formula of the risk coefficient is as follows: risk score = (Exprgs1 × Coefrgs1) + (Exprgs2 × Coefrgs2) + … + (ExprgsN × CoefrgsN).

### Survival difference and prognosis evaluation of high and low-risk groups

Survival analysis and prognosis evaluation were performed for high-risk and low-risk groups through the Kaplan Meyer method and ROC curve analysis.

### Immune infiltrates analysis

TIMER is an online website that can evaluate the Infiltration degree of immune cells to various tumors^16^. CIBERSOFT can use a deconvolution-based algorithm to calculate the abundance of 22 immune cells in different tissues^17^. The data of LUAD related immune cell infiltrates score in TCGA are mainly downloaded from the CIBERSOFT algorithm on the TIMER website.

### Functional enrichment analysis

David 6.8 bioinformatics resources and gene set enrichment analysis (GSEA) were used to perform GO and KEGG function enrichment analysis, respectively. The screening criteria of the GO pathway were *p*-value < 0.05, KEGG pathways with the following criteria were regarded as nominal *p*-value < 0.05.

### Construction and evaluation of a predictive nomogram

Univariate and multivariate Cox regression analysis selected the most significant prognostic factors independently. It established a nomogram to predict the survival probability of one to three years, and the calibration value can be used to predict the prediction accuracy of the nomogram.

### Cell source and culture environment

Lung adenocarcinoma cell lines were mainly purchased from the cell bank of Chinese Academy of Sciences (Shanghai, China), and cultured in RPMI-1640 medium (Gibco, Gaithersburg, MD, USA) with 10% fetal bovine serum (FBS) at 37 °C and 5% CO2.

### Simulated cell hypoxia environment

The cells were planted overnight in a six well plate with a cell density of about 60–70%. The next day, 200 µ mol CoCl2 was added to each well. After 24–36 h, the protein was extracted and WB experiment was carried out. In the cell culture system, cobalt chloride (CoCl2) is a substance that induces cell hypoxia. CoCl2 inhibits the catalysis of proline hydroxylase so that the cells are in a state similar to hypoxia^18–20^.

### Western blotting and RT qPCR experiment

The cells were cultured for 24 h, 1 ml Trizol was added, fully mixed and dissolved, operated according to the kit, and analyzed by RT-PCR. The cells were treated with complete protease inhibitor and Ripa lysate to extract protein. The protein concentration was obtained through the protein quantitative kit. Then, the electrophoresis instrument was modulated and stabilized at 70 V and 110 V for 90 and 60 min respectively, and the target protein was separated. Then, the steady flow 220A was modulated and the membrane was turned for 120 min. Then, the whole membrane was sealed with rapid sealing solution for 30 min. The ratio of working solution of primary antibody and secondary antibody was 1:1000 and 1:3000 respectively. Similarly, The incubation time of the target PVDF membrane is 12 h at 4 °C, and 2H at room temperature respectively. In the middle, the membrane needs to be washed three times for 10 min each time. Due to the small scope of cutting the PVDF membrane, the target antibody may be located at the boundary, but it has little effect on the overall result.

### Statistical analysis

The “survivalroc” package of R (version 4.1.2) was used to analyze the ROC curve, and the “survivminer” and “glmnet” were combined for univariate, multivariate, lasso cox regression and survival difference analysis. The “RMS” package was used to draw the nomogram and calibration diagram, and then the “limma” package was used to analyze the difference of gene expression data. Other R packages that draw graphs of biological information related differences and correlations, including “ggpubr”, “pheatmap”, “ggplot2”, “ggpubr”, “ggextra” and “corrplot” packages in R software. Graphpad prism 8 is used to analyze and draw WB and PCR results.

### Ethics approval

The data used in this study were obtained from publicly available datasets, such as the GEO database (HTTPS:// www. NCBI. nlm. nih. gov/ geo), and The Cancer Genome Atlas (HTTPS:// portal.GDC. cancer. gov). The KEGG and go pathway analysis used was from David database (https://david.ncifcrf.gov/summary.jsp).

## Result

### Determine the most significant independent prognostic clinical factors

**Figure 1:**
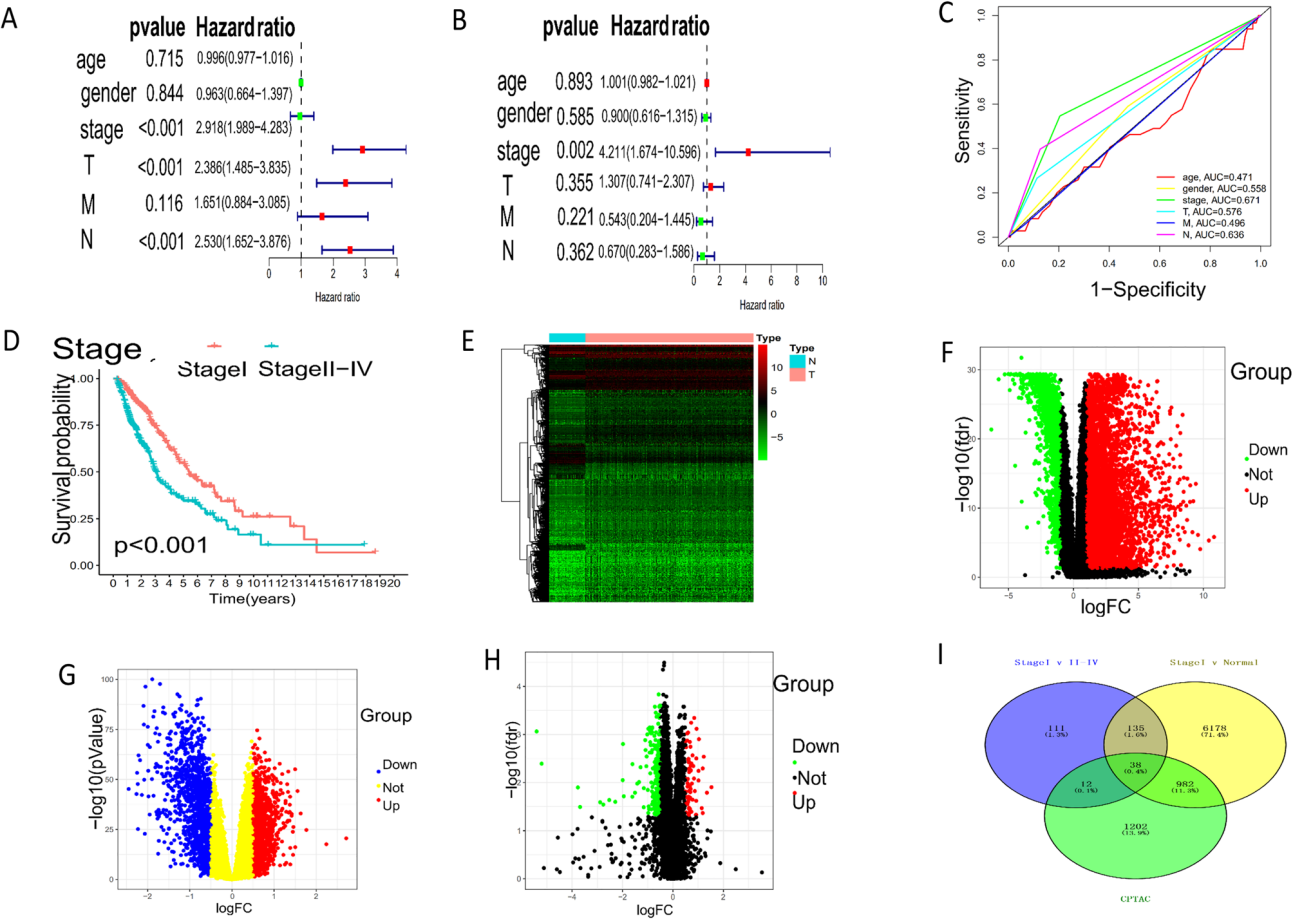
Screening early diagnostic genes related to TMNStage. (**A**, **B**), Forest plots showed univariate (**A**) and multivariate Cox (**B**) analysis of clinical characteristics. (**C**) ROC curve plots of clinical features. (**D**) Survival analysis between stage I and stage II-IV groups. (**E**, **F**) Heat, and (**F**) volcano map of differentially expressed genes between StageI and normal groups from the TCGA database. (**G**) Based on the Cptac database, the heat map showed the differentially expressed proteins between the normal and tumor group. (**H**) Based on the TCGA database, Heatmap showed the differentially expressed genes between StageI and II-IV. (**I**) Wayne’s diagram indicates that early diagnosis genes are screened via the overlaps of differential genes of the three groups.

In univariate and multivariate analysis, we found that only the *p* values of TMN Stage were all less than 0.05 (Fig. 1A, B). The AUC values of various clinicopathological factors (age, gender, TMN stage, T stage, M stage, and N stage) were 0.471, 0.558, 0.671, 0.576, 0.496, and 0.636, respectively (Fig. 1C), and the AUC value of TMN Stage was the highest. They are divided into two groups according to the TMN StageI and TMN StageII-IV. The survival rates of the two groups were significantly different, the prognosis of TMN StageII-IV was worse than that of other groups (*p* < 0.001) (Fig. 1D).

### Screening early diagnostic genes

Based on the normal and Stage groups in TCGA data, we used the Wilcoxon test method in R language to screen 7333 significant genes (Fig. 1E,F), and then differentially expressed between StageI and StageII-IV groups to obtain 296 significant genes (Fig. 1H). We also used the limma package in R language to analyze the differences between normal and tumor groups in the CPTAC database, To obtain 2234 significant genes (Fig. 1G). Finally, 38 early diagnosis genes (edgs) were obtained by the intersection of the above three groups of significant genes (Fig. 1I).

### Screening early diagnosis and prognosis genes and establishing an early prognosis model

**Figure 2:**
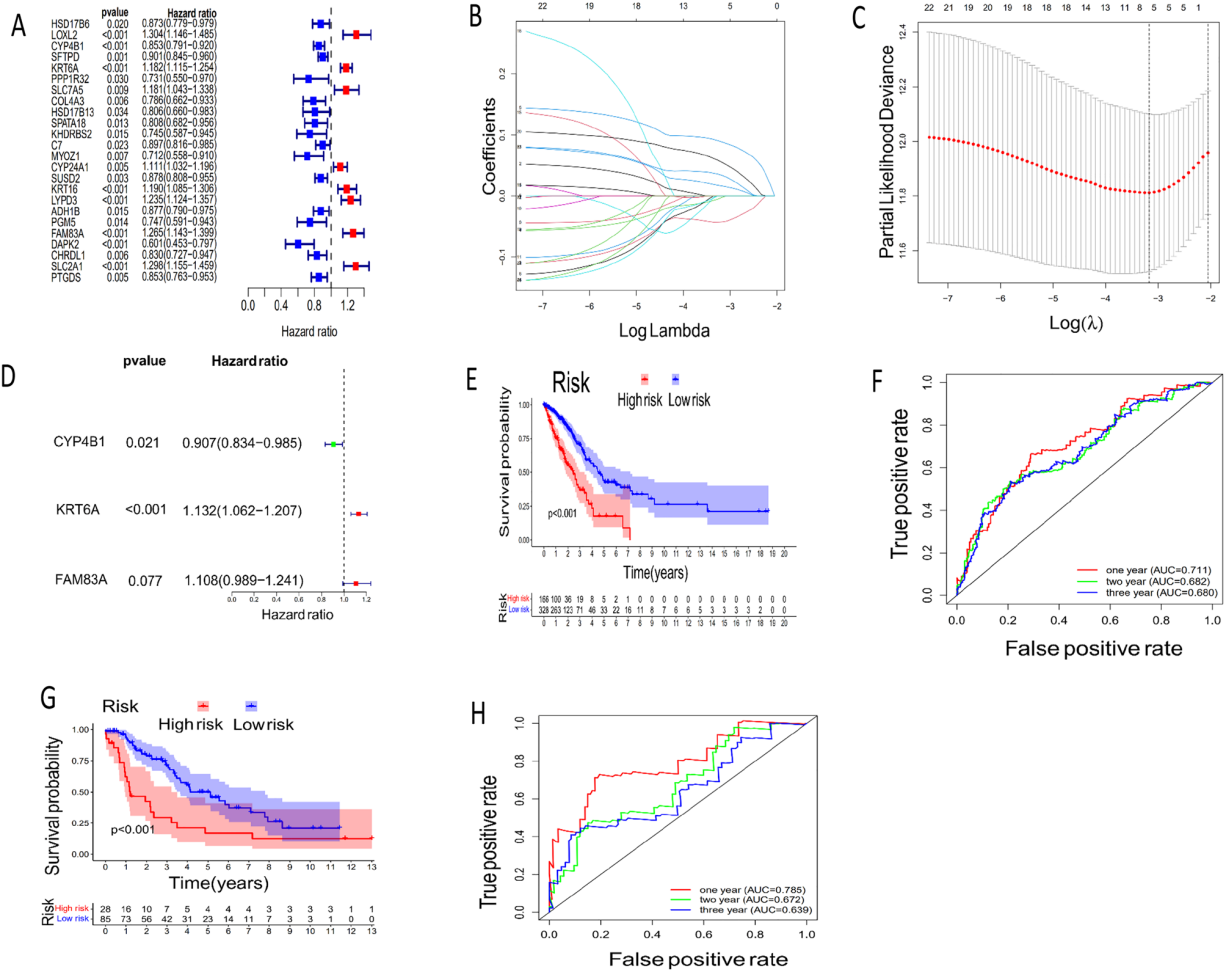
Establish and verify the early diagnosis and prognosis model. (**A**) Univariate Cox survival analysis was performed for the early diagnosis genes. (**B**) A 1000-fold cross-validation for tuning parameter selection in the least absolute shrinkage and selection operator (LASSO) model. (**C**) LASSO coefficient profiles of the 24 early diagnosis and prognosis-related genes. (**D**) Multivariate Cox regression of three risk genes. E, G, KM survival analysis was performed to determine survival differences between different analysis groups, from TCGA and GEO (**G**) data sets. (**F**, **H**) The ROC curve revealed the AUC value of the prognostic model in the TCGA (**F**) and GEO (**H**) cohort.

Firstly, 24 prognostic related edgs (edgps) (Fig. 2A) were screened by univariate Cox regression analysis, then, three early diagnostic markers (risk genes) with the greatest impact on prognosis edgps (Fig. 2B–D) were screened and calculated as risk scores based on lasso Cox regression dimensionality reduction and multivariate Cox regression analysis, to establish early prognostic risk model (edgpsl)^16^. The calculation formula of risk score is as follows: risk score = (−0.09 × Expression CYP4B1) + (0.1238 × Expression value of KRT6A) + (0.1022 × Expression value of FAM83A). Through the “survival” package in R language, the best threshold of clinical prognosis is selected from the risk score, which is used as the basis for the high-risk and low-risk groups. Through KM survival analysis, the study found that the prognosis of high-risk groups is very low, compared with the low-risk group in TCGA (Fig. 2E) and GEO (Fig. 2G) data sets. The AUC value of the three-year prognosis model of the two data sets (Fig. 2F,H) (TCGA: IYAUC = 0.711, 2YAUC = 0.682, 3YAUC = 0.680, GEO: IYAUC = 0.785, 2YAUC = 0.672, 3YAUC = 0.639) were analyzed based on the ROC curve in R soft.

### Correlation between risk score and clinical features

**Figure 3:**
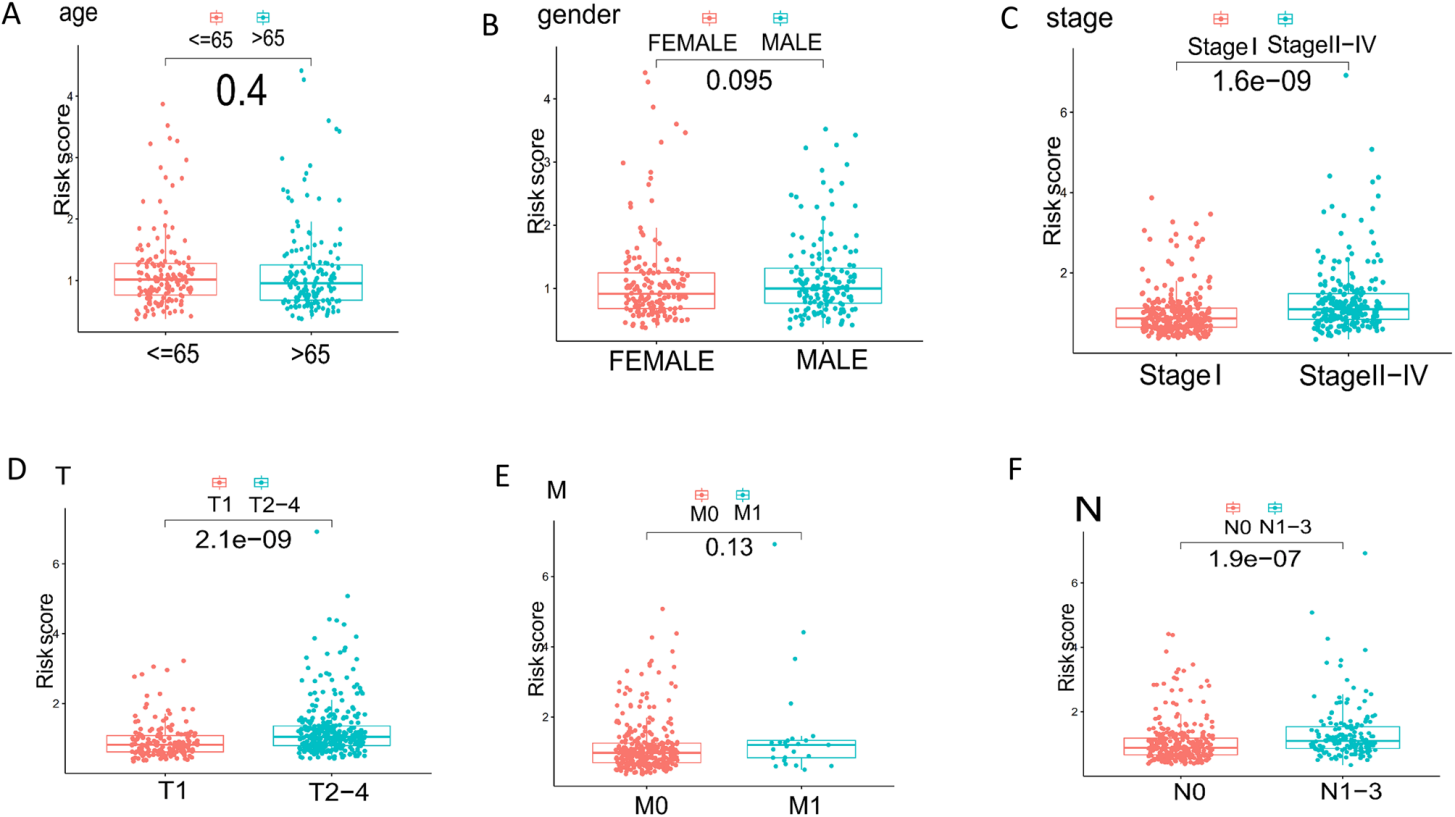
The relationships between risk score and multiple clinicopathological parameters. (**A**) Age. (**B**) Gender. (**C**) TMNStage. (**D**) Tumor size (T). (**E**) Distant metastasis (M). (**F**) Lymph node status (N).

We found that the risk scores were mainly distributed in the T2-4 Stage (Fig. 3D), N1-3 Stage (Fig. 3F), and TMN StageII-IV (Fig. 3C). However, there were no significant differences in the distribution of risk scores among age (Fig. 3A), gender (Fig. 3B), and M groups (Fig. 3E), compared with other clinical Stages.

### Distribution of immune cell infiltration under risk model

**Figure 4:**
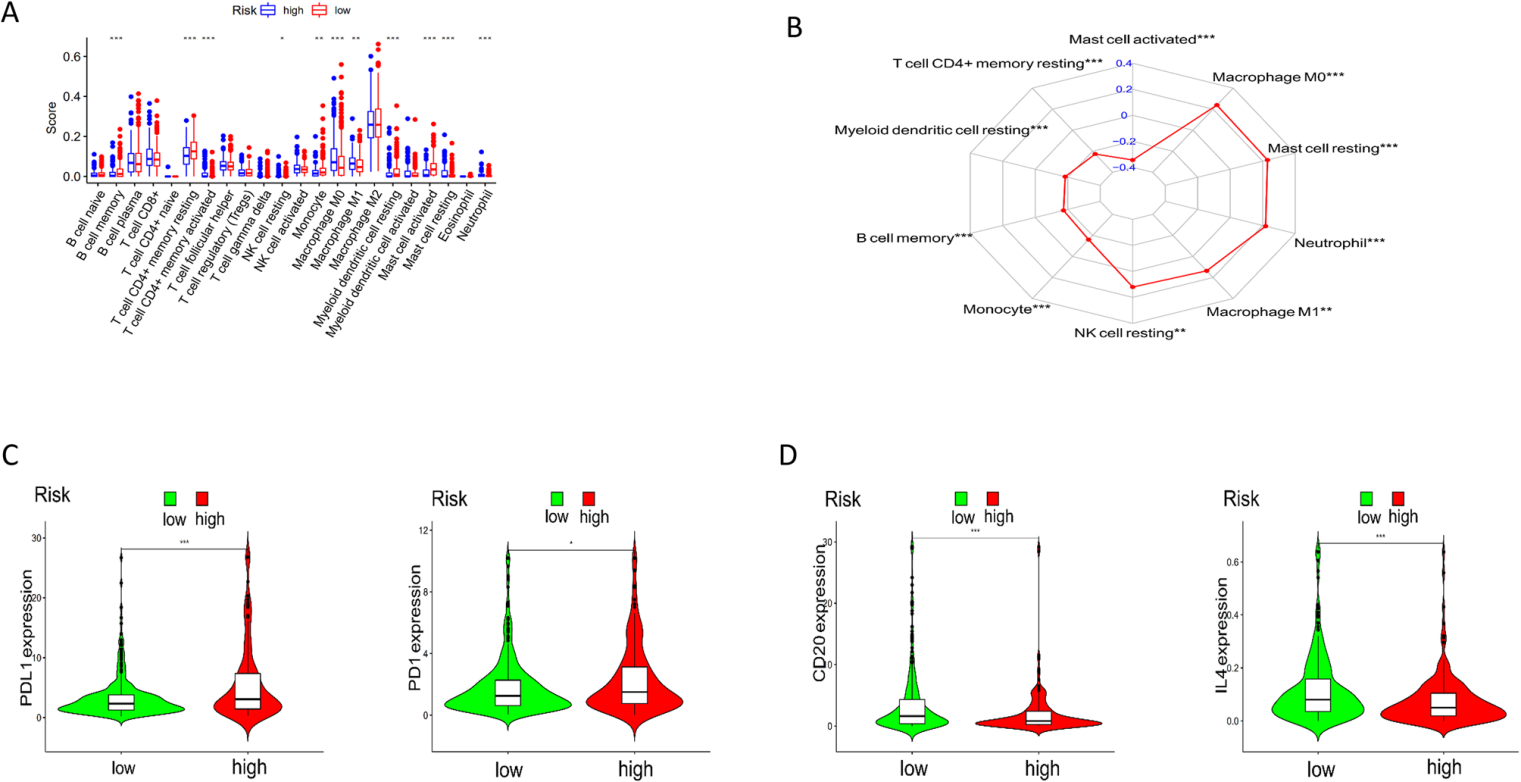
Compositions of infiltrated immune cells between different risk groups in TCGA-LUAD cohort. (**A**) The box plot shows the ratio differentiation of 22 kinds of immune cells between LUAD tumor samples with the high/low risk groups. (**B**) The radar chart shows the correlation between risk score and immune cell score. (**C**, **D**) Violin diagram shows the distribution level of immune related genes in high and low expression groups.

Through this study, we found that 10 immune cells were closely related to the risk score (Fig. 4B), including mast cell activated (cor = −0.34360408), T cell CD4 + memory resetting (cor = −0.226), Myeloid dendritic cell resting (cor = −0.185), B cell memory (cor = −0.174), Monocyte (cor = −0.16), Neutrophil (cor = 0.218), Mast cell resting (cor = 0.23), Macrophage M0 (cor = 0.239), NK cell resting(cor = 0.12) and Macrophage M1 (cor = 0.136). Then, by analyzing the difference of immune cell composition between high and low-risk groups (Fig. 4A), we found that NK cell resting Macrophage M1, Neutrophil, Mast cell resting, macrophage M0 and T cell CD4 + memory activated were mainly distributed in the high-risk group, while the immune cells mainly distributed in the low-risk group were mast cell activated, T cell CD4 + memory resting, myeloid dendritic cell resting, B cell memory, and Monocyte. Finally, compared with the low-risk group, the expression of immune-related genes programmed death-ligand 1 (PD-L1) and programmed cell death protein 1(PD1) were higher in the high-risk group (Fig. 4C). In the low-risk group, the expression levels of interleukin-4 (IL-4) and surface molecule CD20 (CD20) were higher than those in the high-risk group (Fig. 4D).

### Hypoxia effects in the risk model

**Figure 5:**
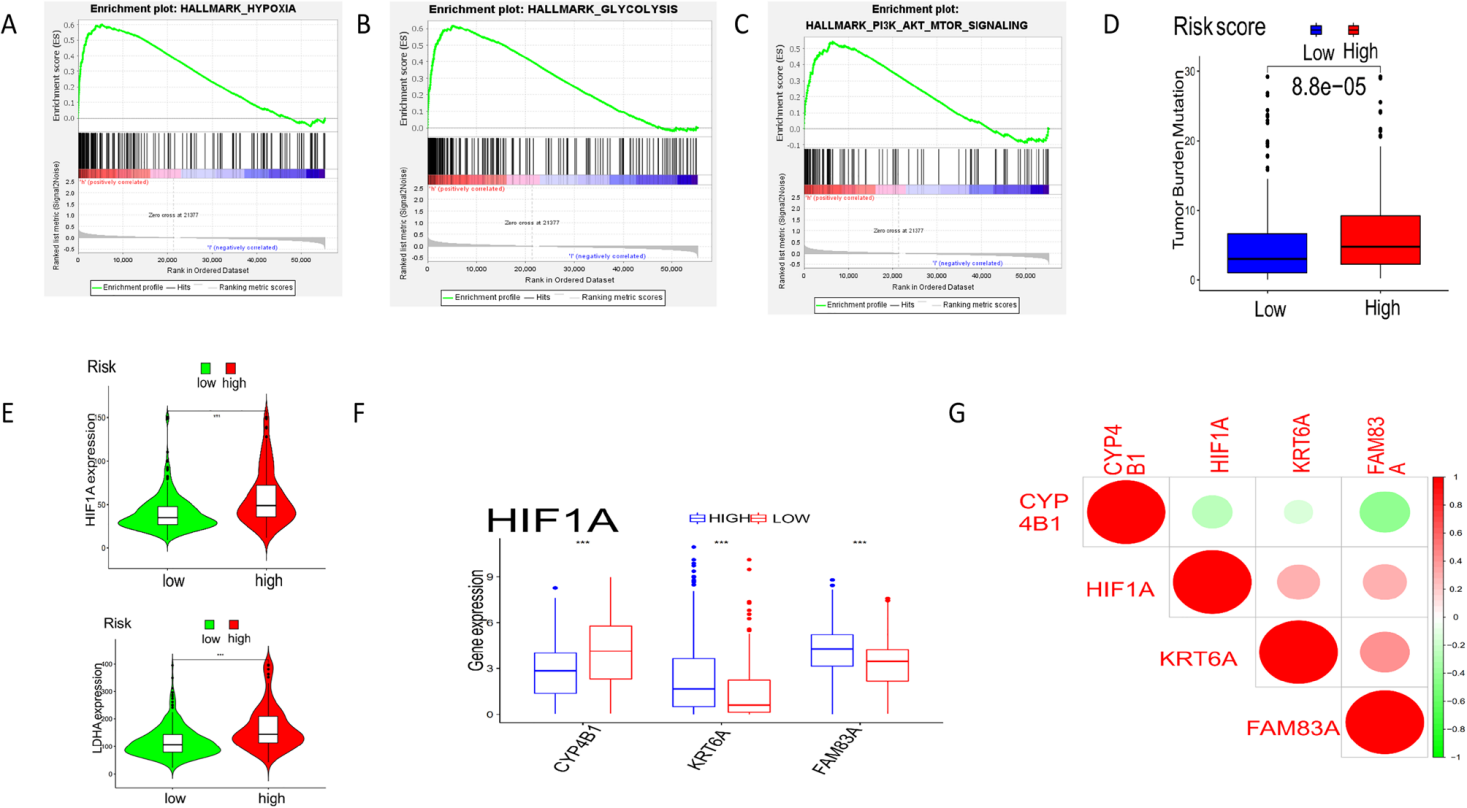
Effects of hypoxia in high and low-risk groups. (**A**–**C**), GSEA analysis showed that the high-risk group was enriched in hypoxia (**A**), glycolysis (**B**), and PI3K/Akt/mTOR (**C**) signaling pathway. (**D**) Tumor mutation load in different risk groups. (**E**) Expression levels of hypoxia-inducible factor HIF1A and hypoxia regulatory factor LDHA. (**F**) Distribution level of risk genes in high and low HIF1A expression groups. (**G**) Correlation between risk genes and HIF1A.

Based on the HALLMARK data set in the GSEA tool, we analyzed the functional enrichment of high and low-risk groups under the risk model. The high-risk group was mainly enriched in hypoxia, glycolysis, and PI3K-Akt-mTOR signal pathway (Fig. 5A–C). The mutation load of the high-risk group was higher than that of the low-risk group (Fig. 5D). At the same time, hypoxia-inducible factor A (HIF1A) and lactate dehydrogenase A (LDHA) were higher in the high-risk group than in other groups (Fig. 5E). In the high HIF1A group, the expression levels of FAM83a and KRT6A were higher, while CYP4B1 was the opposite (Fig. 5F). In addition, FAM83A and KRT6A were positively correlated with HIF1A, while CYP4B1 was contrary (Fig. 5G).

### Establish and verify nomogram based on TMN Stage and risk score

**Figure 6:**
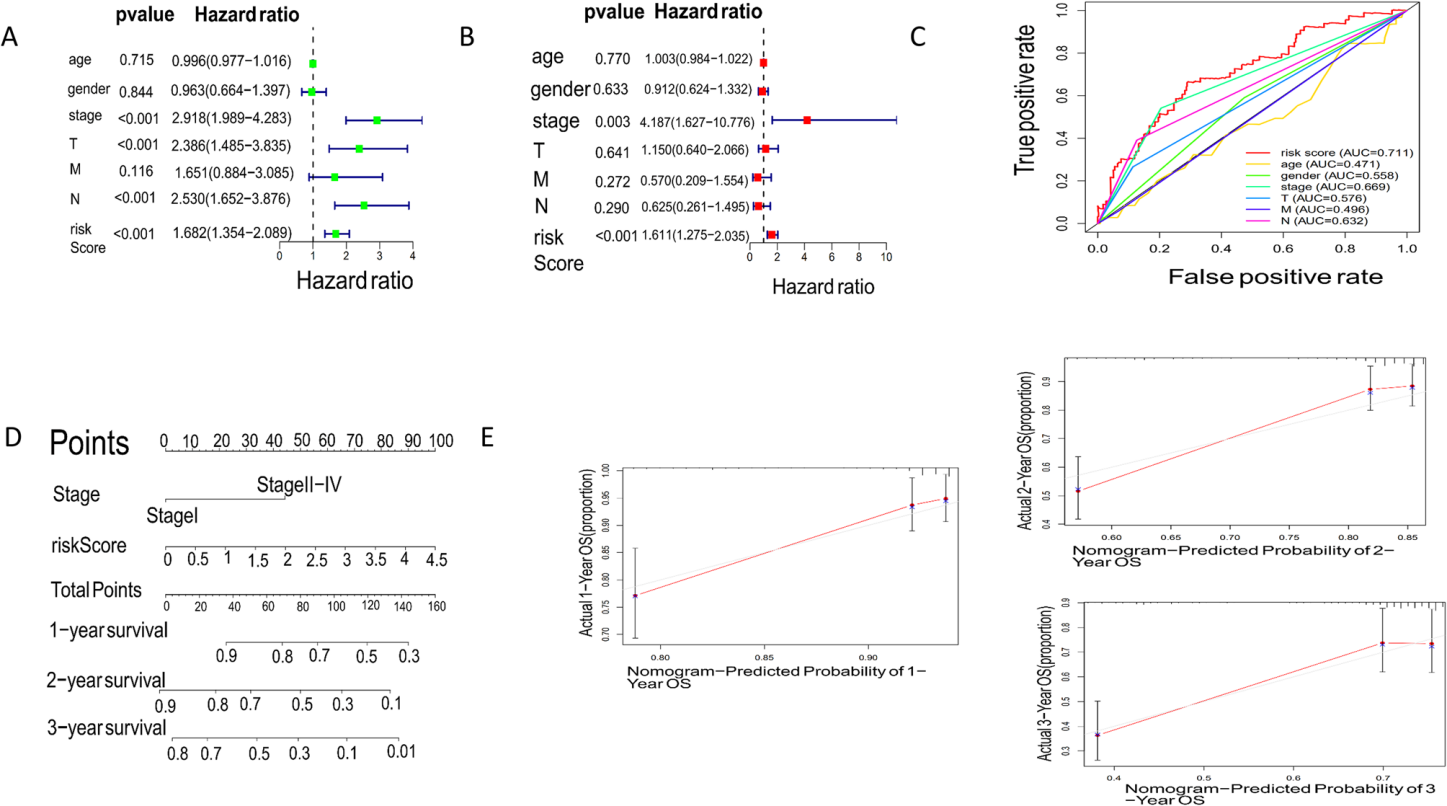
Establishment and evaluation of predictive nomogram in early diagnosis and prognosis model. (**A**, **B**), Forest map showed that independent clinical prognostic factors were obtained by univariate (**A**) and multivariate (**B**) Cox analysis. (**C**) The ROC curve exhibited the predictive performance of each independent predictive factor. (**D**) A nomogram for predicting OS in significantly independent prognostic factors. (**E**) Calibration plot of the nomogram for the probability of OS at 1, 2 and 3 year.

Univariate and multivariate Cox regression analysis of multiple clinical factors and risk scores showed that TMN Stage and risk score had a significant impact on the prognosis of patients. The results of univariate and multivariate analysis showed that TMN Stage (UNIX: HR = 2.918, 95% Cl = 1.989–4.283, *p* < 0.001. Mutiox: HR = 4.187, 95% Cl = 1.627–10.776, *p* = 0.003) (Fig. 6A) and risk score (UNIX: HR = 1.682, 95% Cl = 1.354–2.689, *p* < 0.001. Mutiox: HR = 1.611, 95% Cl = 1.275–2.035, *p* < 0.001) (Fig. 6B). AUC values of various clinical factors and risk scores were analyzed in the "TIMER ROC" package in R language, among which TMN Stage (AUC = 0.669) and risk score (AUC = 0.711) is the highest (Fig. 6C). The nomogram was established based on TMN Stage and risk score to predict the three-year survival rate of patients (Fig. 6D), and the range of C index of 95% confidence interval was 0.6598 to 0.7292. The deviation correction line was consistent with the calibration curve, indicating that the nomogram had high prediction ability (Fig. 6E).

### Functional enrichment analysis of KEGG and GO related to different risk groups

**Figure 7:**
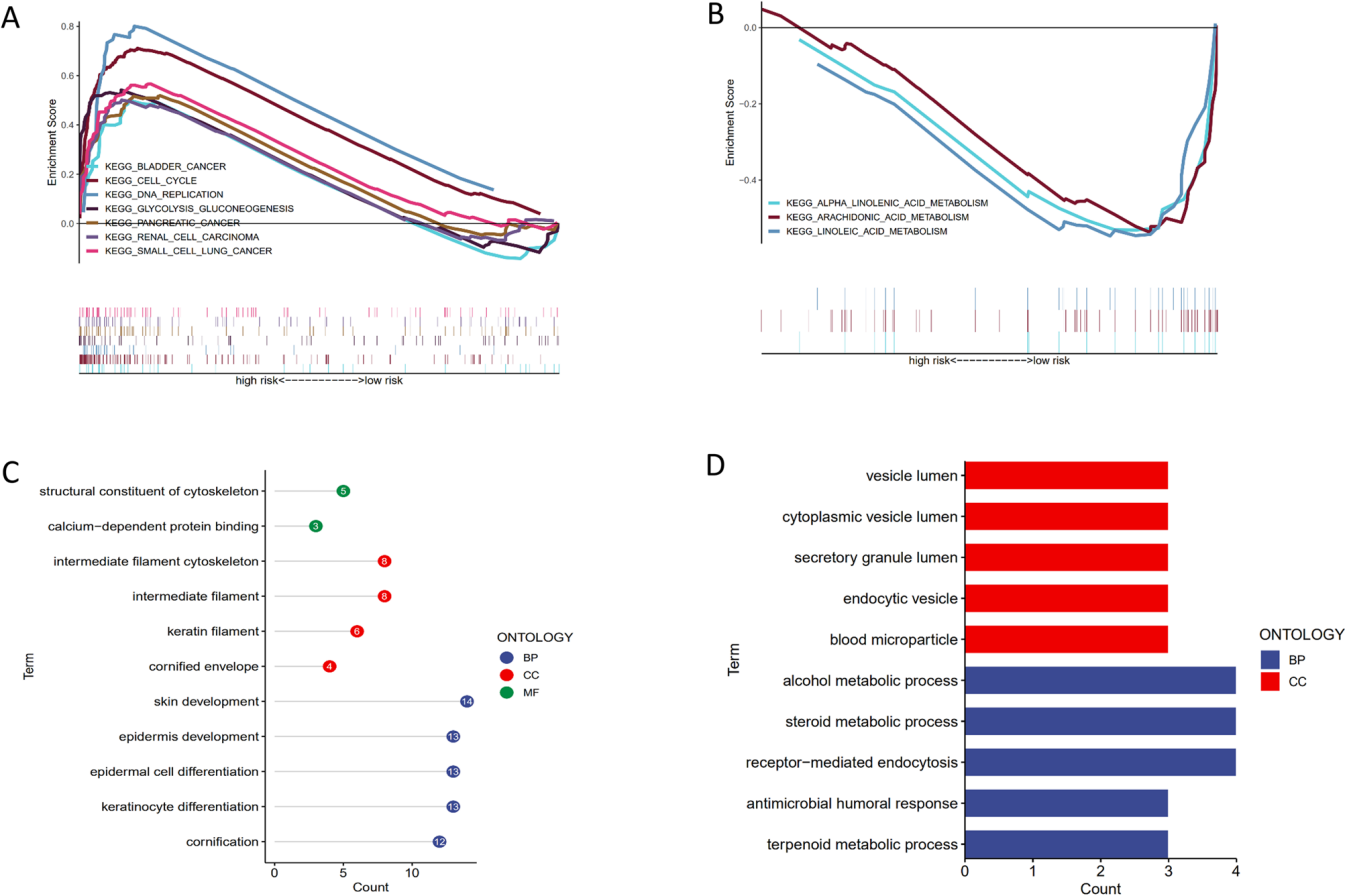
Functional enrichment analysis of KEGG and GO in high and low risk groups. (**A**, **B**), Gene set enrichment analysis (GSEA) results show the enriched KEGG pathways in the high (**A**) and low (**B**) risk groups. (**C**, **D**), Bubbles (**C**) and bar (**D**) plots show enriched go pathways based on up-regulated gene analysis in high-risk and low-risk groups, respectively.

Based on the KEGG data set in GSEA, we found that the low-risk group is mainly enriched in α-Linolenic acid metabolism, arachidonic acid metabolism, and Linoleic acid metabolism (Fig. 7B). In contrast, high-risk groups were primarily increased in bladder cancer, cell cycle, replication of bladder, pancreatic cancer, renal cell carcinoma, and small cell carcinoma signal pathway (Fig. 7A). GO function enrichment analysis was conducted through the DAVID online website for significant genes in high and low-risk groups. The highly expressed genes in high-risk groups were mainly enriched in skin development, keratinocyte differentiation, epithelial cell differentiation, epidermis development, cornification, intermediate filing, intermediate filing cytoskeleton, keratin filing, and structural constitution cytoskeleton, certified envelope, and calcium-dependent protein binding (Fig. 7C). The significant genes in the low expression group were mainly enriched in the receptor-mediated endocytosissteroid metabolic process, alcohol metabolic process, terpenoid metabolic process, antimicrobial humoral response, blood microparticle, endocytic vesicle, secretory granule lumen, cytoplasmic vesicle lumen, and vesicle lumen (Fig. 7D).

### Univariate Cox regression was used to analyze the survival results of OS, DSS, DFI, and PFI related to early prognostic genes

For the univariate cox analysis of early prognostic genes (FAM83A, KRT6A, and CYP4B1) related to OS, DSS, DFI, and PFI of 32 tumors, we selected the top one to three with the lowest *p*-value in the analysis of tumor prognosis. Firstly, in the analysis of overall survival (OS), FAM83A (*p* = 2.06e-07), KRT6A (*p* = 1.22e-07), and CYP4B1 (*p* = 6.72e-05) had the most significant effect on the overall survival of patients with lung adenocarcinoma. Patients with high expression of FAM83A (HR = 1.283,95% CI = 1.168–1.410) and KRT6A (HR = 1.156, 95% CI = 1.097–1.223) had lower survival, while CYP4B1 (HR = 0.866,95% CI = 0.806–0.929) had the opposite effect. In the prognostic analysis of disease-free interval (DFI), CYP4B1 (*p* = 0.003) and FAM83A (*p* = 0.003) had the highest effect on the disease-free progression of lung adenocarcinoma, while KRT6A (*p* = 0.046) had a higher effect on BRCA. High expression of KRT6A (HR = 1.108, 95%CI = 1.004–1.223) and FAM83A (HR = 1.227, 95%CI = 1.074–1.402) had a poor prognostic progression of BRCA and lung adenocarcinoma, while CYP4B1 had the opposite effect on lung adenocarcinoma (HR = 0.860, 95%CI = 0.778–0.950). In the prognostic analysis of disease-specific survival (DSS), CYP4B1 (*p* = 0.000249), FAM83A (*p* = 5.69E-05), and KRT6A (*p* = 0.001235) had the highest effect on the disease-specific survival of lung adenocarcinoma patients. High expression of KRT6A (HR = 1.125, 95%CI = 1.047–1.207) and FAM83A (HR = 1.288, 95%CI = 1.138–1.457) was associated with poor DSS of lung adenocarcinoma, while CYP4B1 had the opposite effect on lung adenocarcinoma. In the prognostic analysis of progression-free interval (PFI), CYP4B1 (*p* = 6.40e-05) and KRT6A (*p* = 0.0111) had the highest impact on PFI in patients with lung adenocarcinoma, and FAM83A had a higher impact on PFI in KIRC (*p* = 0.0002052). High expression of KRT6A (HR = 1.075, 95% CI = 1.017–1.137) and FAM83A (HR = 2.436, 95% CI = 1.522–3.898) were associated with poor PFI of KIRC, while CYP4B1 (HR = 0.874,95% CI = 0.819–0.934) had the opposite effect on lung adenocarcinoma (Tables 1, 2, 3).

**Table 1:** Prognostic analysis of KRT6A.

Cancer	HR	HR.95L	HR.95H	*p* value
**OS.KRT6A**				
**LUAD**	**1.157898871**	**1.096684237**	**1.222530378**	**1.22E-07**
SKCM	1.12564691	1.07449232	1.179236876	6.11E-07
KIRC	1.33188226	1.04838988	1.692033077	0.018928041
UVM	1.805972573	1.069215536	3.050401744	0.027090778
KICH	20,994,725.68	0.701724601	6.28136E + 14	0.054904198
**DF1.KRT6A**				
BRCA	1.107863317	1.003598322	1.222960524	0.042235881
**LUSC**	**0.9222515**	**0.850670131**	**0.999856228**	**0.049593677**
PRAD	0.495357964	0.232569008	1.055082593	0.068611204
CESC	0.919288751	0.832046791	1.015678226	0.098091252
READ	1.45780082	0.800555899	2.654634401	0.217741198
**DSS.KRT6A**				
**LUAD**	**1.124796161**	**1.047339069**	**1.207981677**	**0.001235613**
SKCM	1.091936871	1.033976109	1.153146692	0.001574316
COAD	1.222235192	1.064897446	1.402819465	0.004313416
KIRC	1.440215986	1.091704659	1.899984643	0.00986091
BLCA	1.051843967	1.006594974	1.099127017	0.024261653
**PF1.KRT6A**				
**LUAD**	**1.075291347**	**1.016710535**	**1.137247468**	**0.011091894**
KICH	1743.501268	4.570435971	665,099.9359	0.013853744
COAD	1.152795979	1.028203763	1.292485611	0.014827135
LGG	0.656928963	0.447592771	0.964170315	0.031845272
LUSC	0.940682523	0.889335749	0.99499386	0.032744173

**Table 2:** Prognostic analysis of FAM83A.

Cancer	HR	HR.95L	HR.95H	*p* value
**OS.FAM83A**				
**LUAD**	1.283267168	1.167995496	1.409915218	2.06E-07
SKCM	1.352260012	1.163059339	1.572238903	8.70E-05
KIRC	2.04667282	1.342800827	3.119501828	0.000866229
PCPG	4.264086206	1.629993348	11.15491127	0.003119186
SARC	3.072568146	1.394105096	6.771853169	0.005369308
**DFI.FAM83A**				
**LUAD**	1.227003297	1.073621962	1.402297218	0.002676747
READ	0.000389085	5.41E-08	2.797917423	0.083115389
ACC	0.083825242	0.0031269	2.247168764	0.139562074
UCEC	1.19874334	0.930916679	1.543624288	0.159994472
KIRC	3.70E-15	1.68E-36	8,139,569.677	0.1850627
**DSS.FAM83A**				
cancer	HR	HR.95L	HR.95H	pvalue
KIRC	2.531749356	1.667806837	3.843223724	1.29E-05
**LUAD**	1.288063243	1.138692124	1.457028536	5.69E-05
PCPG	4.831103758	1.795182877	13.00121777	0.001818522
KIRP	1.508219359	1.12146761	2.028347154	0.006561952
KICH	2.83E + 48	4,811,187,490	1.67E + 87	0.01430812
**PFI.FAM83A**				
KIRC	2.436038522	1.522442751	3.897869838	0.0002052
**LUAD**	1.16673582	1.069596073	1.272697711	0.000507183
PCPG	3.223118968	1.332024068	7.799030158	0.009435092
UCEC	1.235075203	1.052890189	1.448784281	0.009516633
KICH	1.04E + 30	9,432,581.341	1.15E + 53	0.010673369

**Table 3:** Prognostic analysis of CYP4B1.

Cancer	HR	HR.95L	HR.95H	*p* value
**OS.CYP4B1**				
**LUAD**	0.865721226	0.806466968	0.929329124	6.72E-05
CESC	0.825741895	0.713579161	0.95553474	0.010152
MESO	0.779690106	0.631823923	0.962161512	0.020368899
THCA	0.639830154	0.417992003	0.979403011	0.039804147
LUSC	1.090883883	1.002476902	1.187087347	0.043660617
**DFI.CYP4B1**				
**LUAD**	0.859734583	0.778129218	0.949898212	0.002976928
PCPG	8.205218674	0.890255101	75.62508028	0.063257081
KIRP	1.277269698	0.962926137	1.694229514	0.089533782
PRAD	0.72483409	0.492999533	1.065689566	0.101747338
MESO	0.494116267	0.19249598	1.268342775	0.142719861
**DSS.CYP4B1**				
**LUAD**	0.841645907	0.76746892	0.922992206	0.000249952
MESO	0.633312562	0.456296813	0.878999786	0.006313294
THYM	2.644638765	1.213617164	5.76303171	0.014400322
CESC	0.826689258	0.698557532	0.978323328	0.02675934
HNSC	0.833749859	0.683911075	1.016416977	0.072040691
**PFI.CYP4B1**				
**LUAD**	0.87428995	0.818558134	0.933816287	6.40E-05
THYM	1.932663954	1.318276644	2.833388557	0.000736535
MESO	0.771820846	0.61397792	0.970242413	0.026503547
BLCA	0.948915708	0.898158471	1.002541366	0.061557204
CESC	0.879924992	0.766162661	1.010579126	0.070144092

**Figure 8:**
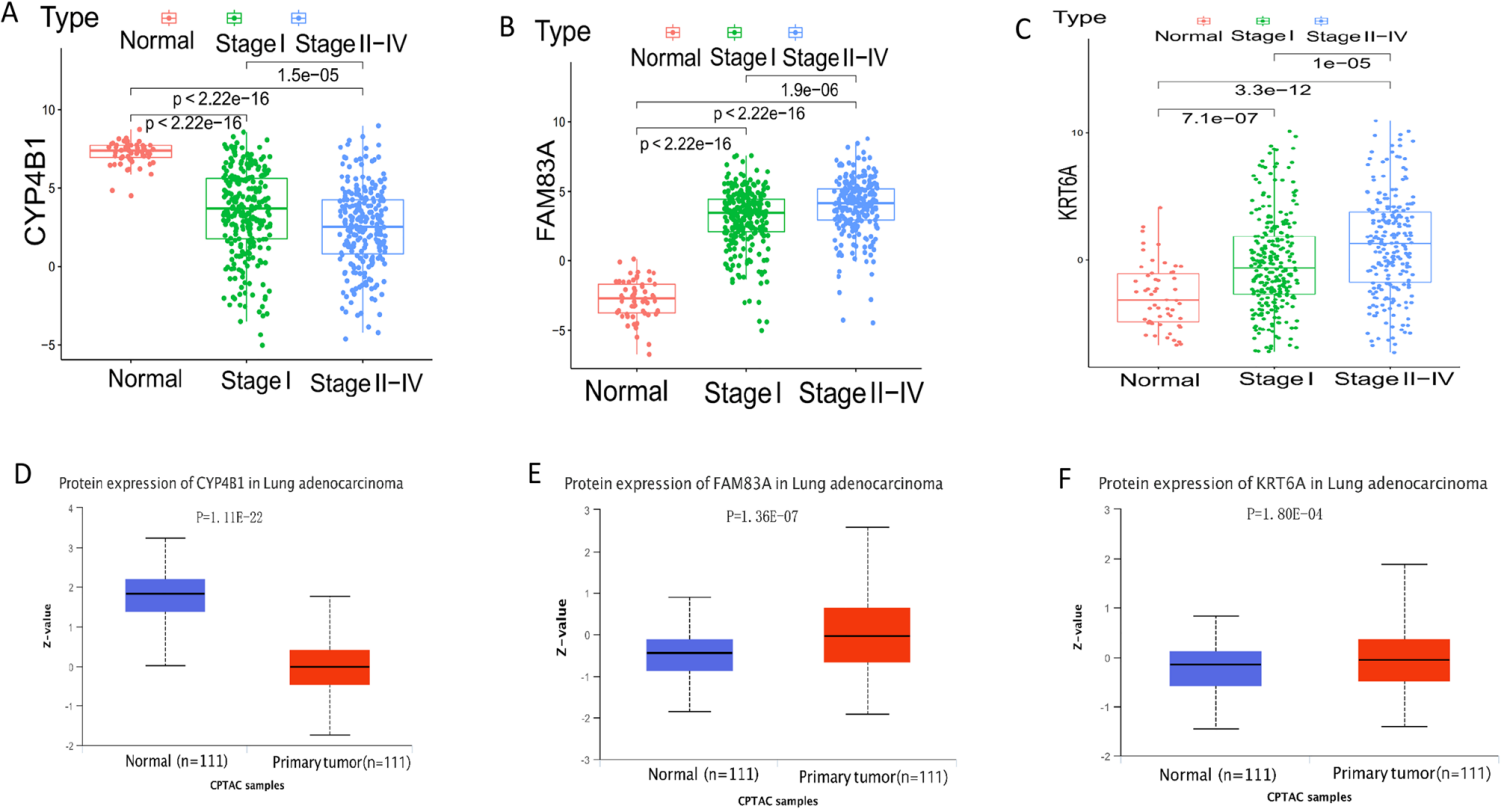
Prognosis and expression results of early diagnosis and prognosis genes in lung adenocarcinoma and other tumors. (**A**–**C**), CYP4B1 (**A**), FAM83A (**B**), and KRT6A (**C**) were expressed in the normal group, stage I, and stage II-IV groups. (**D**–**F**), The expression levels of CYP4B1, FAM83A, and KRT6A in normal and tumor groups were analyzed by using the CPTAC data set in UNLCAN database.

**Figure 9:**
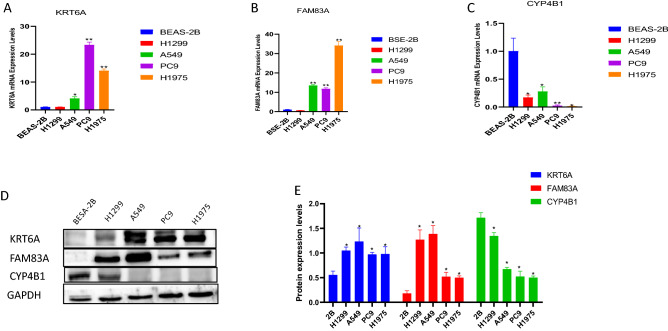
Expression levels of risk genes in normal and tumor cells. (**A**–**C**), MRNA expression levels of KRT6A (**A**), FAM83A (**B**) and CYP4B1 (**C**) in normal and lung adenocarcinoma cells. (**D**, **E**), Protein expression level of risk genes.

Compared with normal and StageI groups, KRT6A and FAM83A were higher in StageII-IV groups, while CYP4B1 expression levels were opposite (Fig. 8A–C). Similarly, in the analysis of the CPTAC dataset in the UALCAN database, the protein expression levels of KRT6A and FAM83A were higher in the tumor group, while CYP4B1 was the opposite (Fig. 8D–F). The mRNA and protein expression levels of FAM83A and KRT6A in tumor cells A549, PC9, and H1975 were higher than those of normal cells BSE-2B, while the mRNA and protein expression level of CYP4B1 in normal cells BSE-2B was higher than that of tumor cells H1299, PC9, A549 and H1975 (Fig. 9A–E) (*p < 0.05, **p < 0.01, ***p  < 0.001).

### Correlation between risk genes and HIF1A in a hypoxic environment

**Figure 10:**
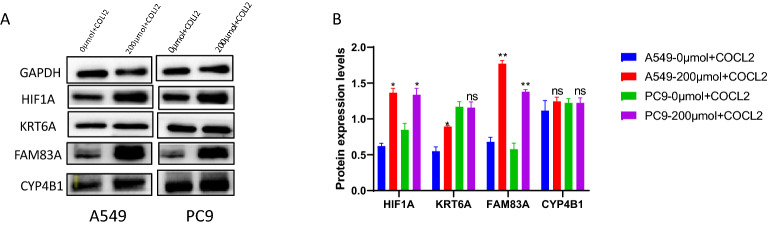
(**A**, **B**) CoCl2 induced hypoxia and normal environment, the expression level of risk genes (KRT6A, FAM83A and CYP4B1) and HIF1A.

HIF1A increased significantly after CoCl2 induced A549 and PC9 cells. Meanwhile, in A549 cells, KRT6A and FAM83A increased with HIF1A. In PC9 cells, only FAM83A increased with HIF1A. The changing trend of CYP4B1 is not apparent (Fig. 10 A-B).

## Discussion

This study evaluated the prognosis and accuracy of various clinicopathological parameters through the univariate, multivariate Cox regression and ROC curve analysis. In different TMN Stage groups, there was a significant survival difference between Stage I and Stage II-IV groups; TMN Stage has the most noticeable impact on the prognosis of patients. In the past study, we also found that TMN Stage can predict the survival probability of patients^9^. At the same time, LUAD, as a fatal malignant tumor, can lead to the five-year survival rate of patients being still very low^21^. In conclusion, we can improve the prognosis of patients by grouping TMN Stage to obtain new potential early diagnostic and prognostic markers. Thirty-eight early diagnosis genes (edgs) were selected from three groups of genes with significant differences, including StageI v Normal, StageI v II-IV, and CPTAC. Then, based on the univariate, lasso, and multivariate Cox regression analysis, three risk genes were selected from edgps and calculated to establish a risk model. The three risk genes were CYP4B1, FAM83A, and KRT6A, respectively. As an extrahepatic form of cytochrome P450, CYP4B1 is mainly highly expressed in the lung and a small amount in other organs^22^. Decreased CYP4B1 is associated with poor prognosis in patients with bladder cancer^23^. Inhibit CYP4B1 can promote the occurrence of lung adenocarcinoma by preventing metabolism, enhancing DNA replication, and cell cycle activity; when the specific situation is unknown, it needs to be verified by experiments^24^. FAM83A is a member of an 8-member protein family. They have a highly conserved N-terminal domain with the same function and unknown function called the duf1669 domain^25^. FAM83A has been proved to promote the proliferation, invasion, stem cell-like characteristics, and drug resistance of lung cancer, breast cancer, and pancreatic cancer^26–31^. Keratin 6A (KRT6A) is a type II keratin involved in the epimerization of squamous epithelium^32,33^. Recent studies found that KRT6A plays a vital role in cell migration, especially keratinocyte migration. Downregulation of KRT6A expression can inhibit cell invasion and metastasis of nasopharyngeal carcinoma. In lung adenocarcinoma, a high KRT6A level is associated with poor prognosis and can promote the growth and metastasis of lung adenocarcinoma by inducing epithelial-mesenchymal transformation^34–38^.

Interestingly, using OS, DSS, DFI, and PFI related univariate Cox regression analysis in various tumors, we found that FAM83A, CYP4B1, and KRT6A had the most significant impact on the survival and progression of lung adenocarcinoma. In the normal tissue and TMN Stage group, FAM83A and KRT6A were the highest in the TMN Stage II-IV group, while CYP4B1 was the opposite. In normal and tumor cells, FAM83A and KRT6A were expressed higher in most tumor cells than in normal cells, while CYP4B1 was the opposite. In conclusion, the three risk genes significantly impact the early diagnosis and prognosis of patients with lung adenocarcinoma. The patients were divided into high and low-risk groups, according to the risk score in the early prognosis model. In the correlation analysis of multiple clinicopathological indexes and risk scores, we found that the risk scores in T2-4, N1-3, and StageII-IV groups were generally higher than those in other groups. Then, through univariate and multivariate cox regression analysis, we found that TMN Stage and risk score was the more significant independent prognostic factors. The two independent prognostic factors were used to establish a nomogram with good prediction ability and accuracy.

Using the immune score analyzed by the CIBERSORT method on the TIMER website, we found that NK cell resting, macrophage M1, neutrophil, mass cell resting, and macrophage M0 cells were distributed higher in a high-risk group, and the risk score was positively correlated with these immune cells. Mass cell activated, T cell CD4 + memory resting, myeloid dendritic cell resting, B cell memory, and monocyte cells were highly distributed in the low-risk group and negatively correlated with a risk score. Through David's online website's GO function enrichment analysis, the essential immune-related pathway in the low-risk group is a human antimicrobial response; B cell plays a vital role in humoral immunity^39,40^. Interleukin 4 (IL4) and B cell surface receptor CD20, which promote the proliferation and development of B cells, were highly expressed in the risk group^41–46^.

Based on the hallmark data set analysis in the GSEA tool, the high-risk group was mainly enriched in hypoxia, PI3K-Akt -mTOR, and the glycolysis signal pathway. At the same time, the HIF1A, LDHA, and mutation load are higher in the high-risk group than in the risk group. In previous studies, tumors in a hypoxic environment are more likely to lead to poor prognosis and mutations^47,48^. Under hypoxic conditions, HIF1A expression increases and induces downstream signal pathways, and PI3K -Akt-mTOR signaling pathway can promote tumor proliferation^49–53^. Glycolysis can enhance tumor proliferation, and it is a crucial enzyme to promote glycolytic activity^54–56^. Interestingly, HIF1A can also regulate glycolysis through the PI3K-Akt-mTOR signaling pathway^57^. Through the analysis of KEGG data set in the GSEA tool, the high-risk group is mainly enriched in the signal pathways related to cancer, such as boulder cage, cell cycle, DNA replication, glycolysis gluconeogenesis, pancreatic cage, renal cell carcinoma, and small cell lung. The low expression group was mainly enriched in alpha-linolenic acid metabolism, linoleic acid metabolism, and arachidonic acid metabolism pathway. In related learning, we found that alpha-linolenic acid and arachidonic acid metabolism can inhibit tumors and promote apoptosis^58–61^. Finally, through RT-PCR and WB, we found that the expression levels of tumor cells (A549, H1975, and A549) in BESA-2B cells were significantly higher than those in FAM83A and KRT6A, while CYP4B1 was the opposite. In addition, FAM83A and HIF1A are positively correlated, after CoCl2 induced hypoxia. This discovery has opened up new ideas for us.

In conclusion, based on various bioinformatics methods and cytological experiments, the screened risk genes can be used as potential early prognostic diagnostic markers of lung adenocarcinoma, which is also closely related to the development of immunity and hypoxia. This series of findings will provide a new idea for the comprehensive treatment of LUAD.

## Supplementary Information

Supplementary Information.

## Data Availability

Publicly available datasets were analyzed in this study; these can be found in the GEO database (https://www.NCBI.nlm.nih.gov/geo), and The Cancer Genome Atlas (https://portal.GDC.cancer.gov). The authors confirm that the data supporting the findings of this study are available within the article and its Supplementary materials. A statement that all methods and public data are implemented by relevant guidelines and regulations.
