# Supplementary material for: Regulation of early diagnosis and prognostic markers of lung adenocarcinoma in immunity and hypoxia
Source: Sci Rep. 2023 Apr 20;13:6459. doi: 10.1038/s41598-023-33404-8 (PMC10119119; doi:10.1038/s41598-023-33404-8)

FIG9.CYP4B1 (50-70KD)

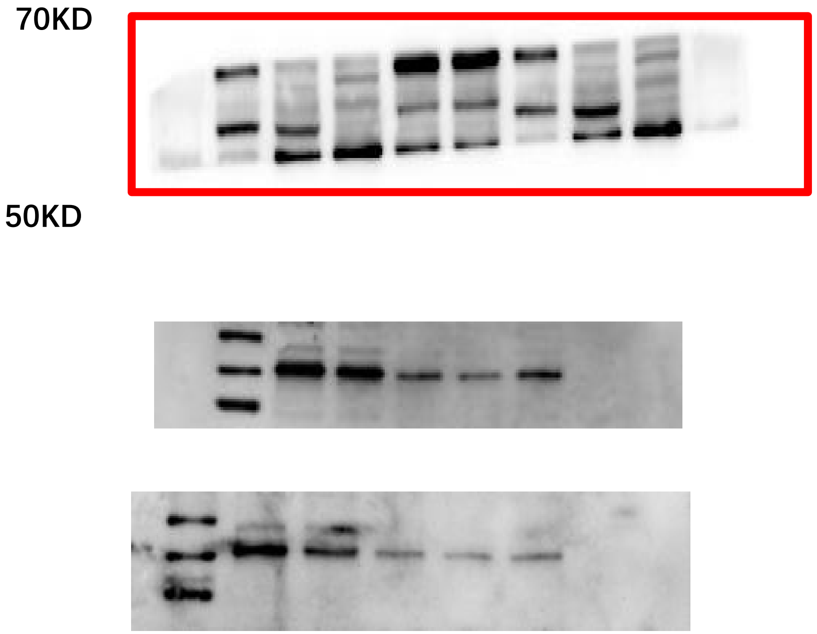

FIG9.FAM83A (40-50KD)

40KD
50KD

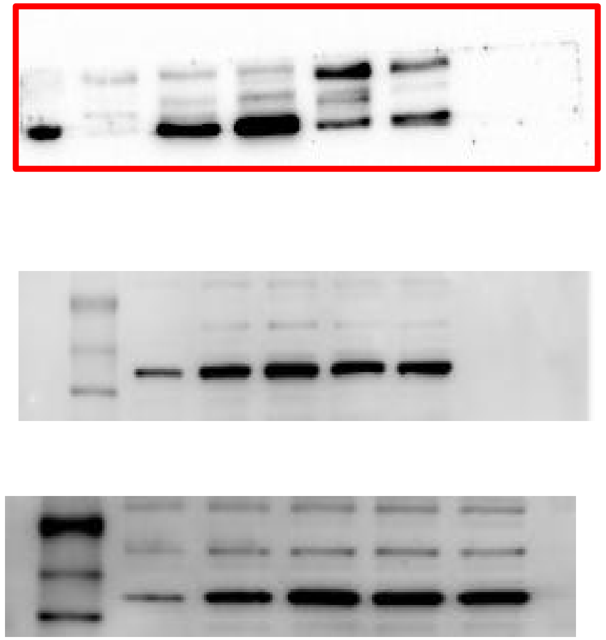

FIG9.GAPDH (25-40KD)

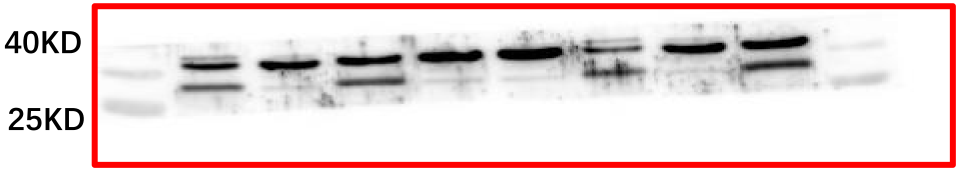

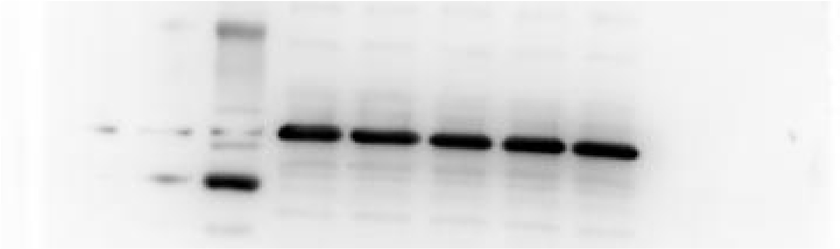

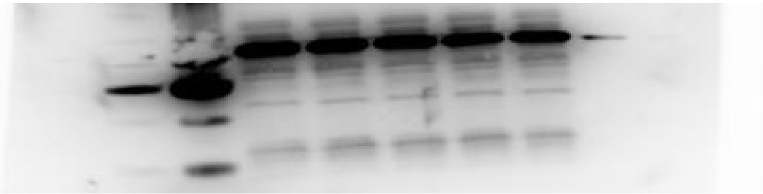

FIG9.KRT6A (50-70KD)

50KD

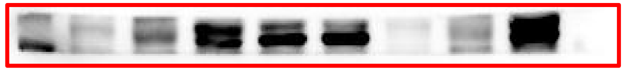

70KD

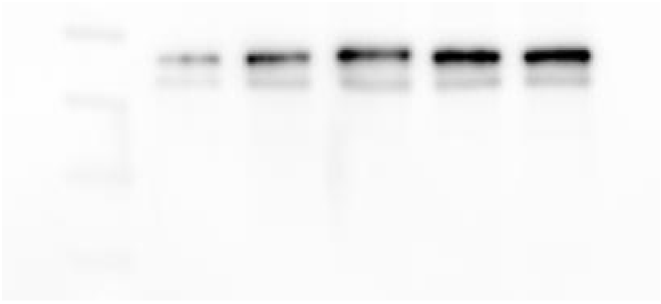

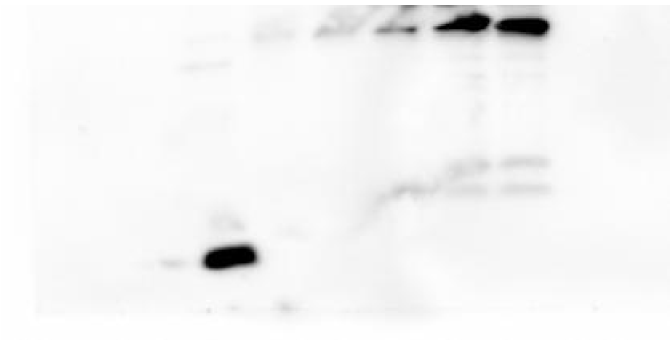

FIG10.CYP4B1
(50-70KD)

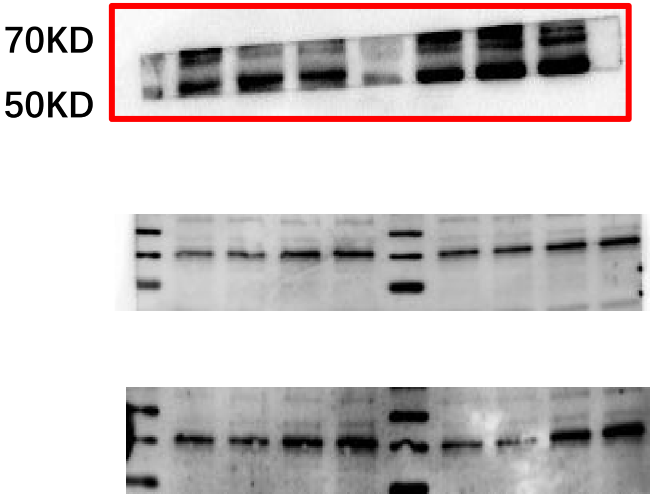

FIG10.FAM83A（40-50KD)

50KD
40KD

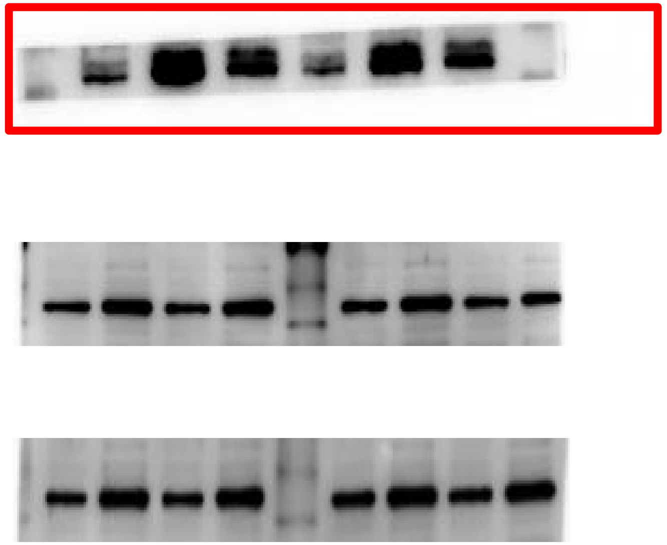

FIg10.GAPDH (35-40KD)

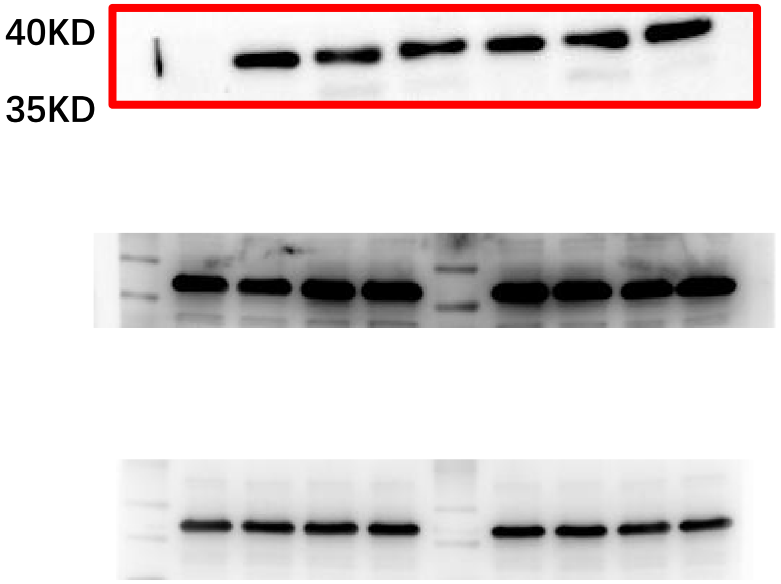

FIG10.HIF1A (100-150KD)

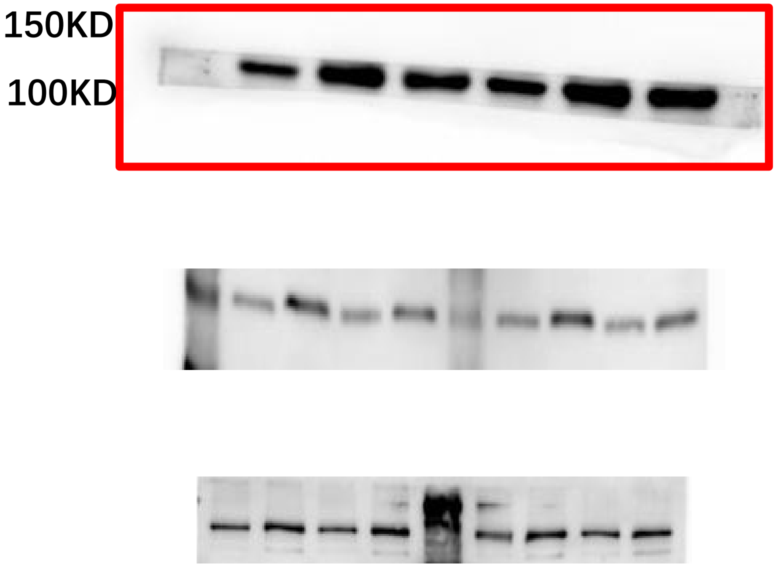

FIG10.KRT6A (50-70KD)

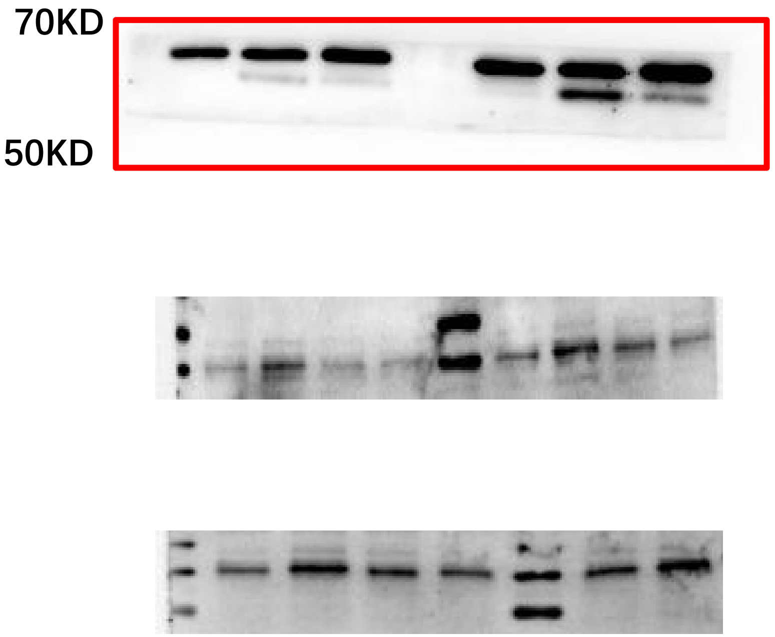

Supplement: Supplementary file 1 — Supplementary Information. [file 41598_2023_33404_MOESM1_ESM.pdf]
